# Prevalence, Diagnosis, and Treatment of Ovarian Cysts in Bitches and Queens: A Meta-Analysis

**DOI:** 10.3390/ani15192800

**Published:** 2025-09-25

**Authors:** Kinga Domrazek, Katarzyna Kondratek, Filip Tobolewski, Piotr Jurka

**Affiliations:** 1 Department of Small Animal Diseases and Clinic, Institute of Veterinary Medicine, Warsaw University of Life Sciences, Nowoursynowska 159c, 02-776 Warsaw, Poland; s202328@sggw.edu.pl (K.K.); piotr_jurka@sggw.edu.pl (P.J.); 2 International Veterinary Students’ Association (IVSA), 02-776 Warsaw, Poland; 3 Actaware, Concord, CA 94518, USA; filip.tobolewski@actaware.com

**Keywords:** ovarian cyst, bitch, queens, meta-analysis, veterinary medicine

## Abstract

The aim of this study was to conduct a systematic review and meta-analysis to determine the prevalence of ovarian cysts in bitches and queens, to classify cyst subtypes, and to evaluate diagnostic and therapeutic strategies reported in the literature. A comprehensive search of PubMed, Scopus, and Google Scholar identified 4321 articles, of which 13 met the inclusion criteria, providing data on 428 bitches and 273 queens. The pooled prevalence of ovarian cysts was 41.7%, with follicular cysts being the most frequently reported subtype. Dogs were significantly more likely to develop cysts than cats, and animals older than five years had a markedly higher risk. Histopathology and ultrasonography were the predominant diagnostic methods, though only one study assessed diagnostic sensitivity. All included studies reported surgical treatment, while non-surgical options and postoperative outcomes were not evaluated. The studies suitable for analysis were also highly variable in reporting, from large studies with near 100% cyst presence in the sample studied, to small studies with relatively low cyst presence, which limits the ability to compute statistical outcomes in a highly reliable way. These findings highlight the high prevalence of ovarian cysts in small animals, particularly in older queens and bitches, and reveal major gaps in standardized diagnostic criteria, non-invasive biomarkers, and therapeutic research. Future prospective studies are needed to validate diagnostic tools, investigate medical management options, and improve evidence-based clinical decision-making in veterinary practice.

## Simple Summary

Ovarian cysts—fluid-filled structures in the ovaries—are a common reproductive issue in female dogs and cats. These cysts can interfere with fertility and cause hormonal problems, yet little is known about how often they occur or how best to detect and treat them. In this study, we reviewed and analyzed data from 13 scientific articles, covering over 700 animals, to estimate how frequently these cysts occur and what diagnostic or treatment methods are being used. We found that ovarian cysts are quite common, especially in older animals, and are more frequently diagnosed in cats than in dogs. However, there is a lack of standard methods for diagnosing these cysts, and nearly all studies focused only on surgical removal of the ovaries. Non-surgical treatment options were rarely discussed, and important diagnostic tools such as hormone testing were underused. Our findings show a need for better research to understand how these cysts develop, how to detect them early, and how to treat them without surgery—especially in animals used for breeding. This could improve animal welfare and help veterinarians make more informed decisions.

## 1. Introduction

Ovarian cysts are fluid-filled structures that develop within or on the surface of ovaries in female dogs (bitches) and cats (queens). They are distinguished into follicular cysts, lutein cysts, cysts of subsurface epithelial structure, cystic rete ovarii, and cystic corpus luteum [1,2,3]. Each type presents distinct histological characteristics and clinical implications. These cystic formations constitute a significant clinical entity in small animal reproduction, untreated often lead to infertility [1,2]. The prolonged presence of ovarian cysts that produce steroid hormones not only increases the risk of uterine disorders but also negatively impacts ovulation, resulting in reduced litter size. The most common uterine histopathologies observed were cystic endometrial hyperplasia (CEH), periglandular fibrosis, lymphoplasmacytic endometritis, and adenomyosis [2].

Ovarian cysts frequently disrupt normal reproductive physiology through hormonal imbalances [4,5]. Follicular cysts, the most clinically relevant type, produce sustained estradiol leading to persistent estrus, prolonged proestrus, and hyperestrogenism with associated complications including cystic endometrial hyperplasia-pyometra complex and bone marrow suppression [2,5]. Luteinized follicular cysts, luteal cysts and cystic corpus luteum, characterized by progesterone hypersecretion, induce mammary gland hyperplasia, and behavioral changes mimicking pregnancy, with associated risks of endometrial pathology and metabolic disturbances like insulin resistance [3,6,7].

Diagnostic challenges in ovarian cyst management arise from their often subclinical presentation and small size (most ≤0.5 cm) [4], often requiring both ultrasonography and hormonal assays for detection. Advanced diagnostic protocols may incorporate vaginal cytology additionally. Histopathological examination remains essential for definitive cyst classification and assessment of structural characteristics. It also enables assessment of endocrine activity through specialized staining techniques and immunohistochemical markers [1,8].

Therapeutic management presents clinical dilemmas between conservative and surgical intervention. While hormonal therapy using gonadotropin-releasing hormone analogs (GnRH) or human chorionic gonadotropin (hCG) has demonstrated success rates of approximately 63% in some studies [2,6], the potential for recurrence and the frequent concurrent presence of uterine pathology often necessitates surgical intervention. Ovariohysterectomy remains to be the most common used treatment [1]. Hormonal therapy challenges are further complicated by the need to exclude pre-existing uteropathies and estrogen-induced hematological changes before attempting conservative management, making comprehensive pre-treatment evaluation essential for optimal patient outcomes [4,7].

Although individual studies have addressed the presence of ovarian cysts in female dogs and cats, the findings remain fragmented and inconsistent. A systematic review and meta-analysis are needed to consolidate current knowledge, quantify the prevalence of ovarian cysts, and identify potential risk factors such as species, breed, and age. Such an analysis may also highlight diagnostic and therapeutic gaps and inform future clinical and research priorities.

The aim of this study was to estimate the prevalence of ovarian cysts in bitches and queens through a systematic review and meta-analysis. Additionally, we aimed to categorize the types of cysts identified, evaluate diagnostic methods and treatment approaches used in the literature, and assess the quality of available evidence.

Therefore, this systematic review and meta-analysis aimed to address the following research question: What is the prevalence of ovarian cysts in bitches and queens, and which diagnostic and therapeutic strategies have been most commonly reported? To answer this question, we applied the PICO framework: the population consisted of female dogs and cats described in clinical and pathological studies; the interventions included diagnostic methods such as ultrasonography, histopathology, and hormonal assays, as well as surgical and medical treatments; the comparisons focused on species differences (dogs versus cats) and diagnostic modalities; and the outcomes were the prevalence and types of ovarian cysts, the accuracy of diagnostic tools, and the reported treatment outcomes.

## 2. Materials and Methods

We followed PRISMA guidelines to perform a systematic review and meta-analysis, focusing on the prevalence, diagnostic approaches, and treatment strategies for ovarian cysts in bitches and queens. The systematic review and meta-analysis were registered in the PROSPERO database under the title: Prevalence, diagnosis, and treatment of ovarian cysts in bitches and queens: a systematic review and meta-analysis.

### 2.1. Study Selection

In the first stage, we conducted a systematic review of ovarian cysts in dogs and cats. After reviewing a large number of publications, we decided to perform a meta-analysis on the prevalence of various types of ovarian cysts in bitches and queens. We followed the PRISMA guidelines as a checklist while preparing the manuscript. The databases used included PubMed, Scopus, and Google Scholar. A comprehensive literature search was conducted in June and July 2025. The keywords used are presented in Table 1.

### 2.2. Inclusion and Exclusion Criteria

Eligible studies were included based on the following inclusion criteria: (a) original research articles reporting data on ovarian cysts in female dogs (bitches) and/or cats (queens); (b) studies published between January 2000 and June 2025; (c) articles written in English; (d) studies providing extractable numerical data (quantitative information allowing calculation of prevalence or proportions) on at least one of the following: prevalence, diagnostic method, treatment, treatment outcomes. The exclusion criteria were as follows: (a) duplicate publications or low-quality studies (e.g., missing raw data, inappropriate methodology); (b) conference abstracts, reviews, commentaries, editorials, or case reports; (c) studies involving other species or mixed-gender groups where data could not be isolated for female dogs or cats; (d) in vitro or experimental laboratory studies without in vivo animal outcomes.

### 2.3. Data Extraction

Screening and data extraction were carried out by one researcher who first conducted literature and data screening according to the criteria developed. Another researcher ensured that the collection of the material was consistent. The established literature screening criteria were as follows: initial screening to exclude articles that clearly did not meet the criteria on the basis of the title and the abstract, followed by a detailed reading of the literature to select the final articles for inclusion in this study on the basis of the inclusion and exclusion criteria. The following data were collected from the included articles: first author, country of study, year of publication, type of sample, detection methods, and results.

### 2.4. Assessment of Study Quality and Risk of Bias

The methodological quality of the included studies was assessed independently by two reviewers using appropriate tools based on study design. For observational studies, the Newcastle–Ottawa Scale (NOS) was applied, evaluating three domains: selection of participants, comparability of study groups, and outcome assessment. Each study could receive a maximum of 9 points, with ≥7 points considered high quality.

In the case of randomized controlled trials (RCTs) (if included), the Cochrane Risk of Bias 2 (RoB-2) tool was used, covering domains such as randomization process, deviations from intended interventions, missing outcome data, outcome measurement, and selection of reported results. Studies were categorized as low, moderate, or high risk of bias.

### 2.5. Statistical Analysis

From each eligible study we extracted the number of animals examined and the number diagnosed with ovarian (or para-ovarian) cysts. Study-specific prevalences were expressed as proportions with 95% Wilson score confidence intervals; a 0.5-animal continuity correction was applied to studies reporting 0% or 100%. Pooled prevalence and its 95% CI were calculated with a DerSimonian–Laird random-effects model; between-study heterogeneity was quantified with τ^2^ and I^2^ statistics. Publication-bias/small-study effects were explored visually with funnel plots (raw proportion and log-odds scales) and formally with Egger’s weighted regression. Species differences (dog vs. cat) were evaluated with Fisher’s exact test, Yates-corrected χ^2^, and by reporting risk and odds ratios. To assess age, we converted reported mean (or mid-range) cohort ages to years and tested the rank correlation between age and prevalence (Spearman ρ) plus a Mann–Whitney U comparison of cohorts ≤ 5 years versus >5 years. Random-effects sub-group meta-analyses were run for each diagnostic modality represented by at least two studies. All computations were performed in Python 3.11.9 (pandas 2.3.1, SciPy 1.16.0, statsmodels 0.14.5) visualizations were performed in matplotlib 3.8.4 and seaborn 0.13.2.

## 3. Results

### 3.1. Characteristics of Included Studies

**Figure 1:**
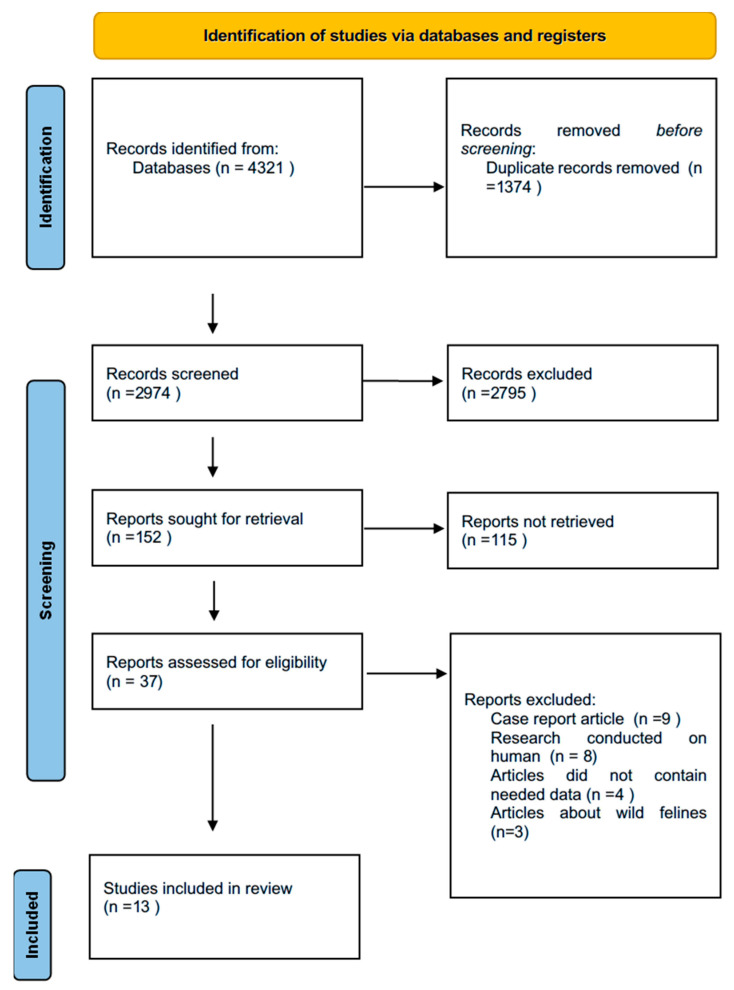
Prisma Flow chart.

Our systematic search across four databases (PubMed, Scopus, and Google Scholar) yielded a total of 4321 articles. After removing 1374 duplicate records retrieved from multiple sources, 2828 articles were excluded based on predefined inclusion and exclusion criteria after screening titles and abstracts. Following full-text assessment, an additional 92 studies were excluded due to insufficient primary data, lack of relevant outcomes, or inappropriate study design, resulting in a final inclusion of 13 articles for quantitative synthesis (Figure 1) [8]. These studies included data on female dogs and cats from various countries and covered different diagnostic approaches (e.g., ultrasound, histopathology). Data extracted from each eligible study were synthesized qualitatively and are summarized in Table 2. No studies addressing clinical aspects of ovarian cysts, such as treatment options or fertility implications, were identified. However, some relevant case reports are available and have been cited when they provide additional insights.

### 3.2. Characteristics of the Study Population

The analyzed studies included a total of 428 bitches and 273 queens. The age of the animals varied across studies. In dogs, the reported mean age ranged from 1.8 to 9.6 years, with individual age ranges extending from 0.3 to 16 years. For cats, age data were less frequently reported, but when available, it ranged from 0.6 to over 13 years. Regarding breed distribution, a wide variety of dog breeds were represented, with the most frequent being German Shepherds (*n* = 53), mixed-breed dogs (*n* = 25), and Labrador Retrievers (*n* = 14). Among cats, the majority were European Shorthairs (*n* = 105), mixed-breed (*n* = 9), and Persian cats (*n* = 20). In several studies, breed data were not specified (dogs: *n* = 194; cats: *n* = 129). The exact number of individuals for each breed is presented in Supplementary Table S1.

### 3.3. Risk of Bias and GRADE Evaluation

**Figure 2:**
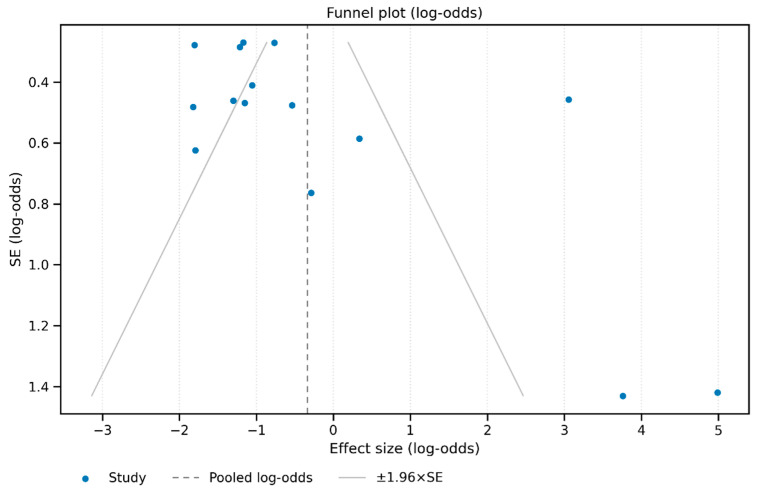
Funnel plot on the log-odds scale. Points are study effects (log-odds) against their standard errors (y-axis inverted). The dashed vertical line marks the random-effects pooled estimate; gray lines show ±1.96 × SE pseudo-confidence bounds. Visual inspection suggests possible asymmetry (small-study effects); Egger’s regression was also performed.

Assessment of publication bias using funnel plots (both log-odds and raw proportion scales) suggests some asymmetry (Figure 2), and Egger’s regression confirmed statistically significant small-study effects (intercept = 3.69, *p* = 0.008), indicating possible publication bias.

### 3.4. Ovarian Cysts

#### 3.4.1. Prevalence of Ovarian Cysts

**Figure 3:**
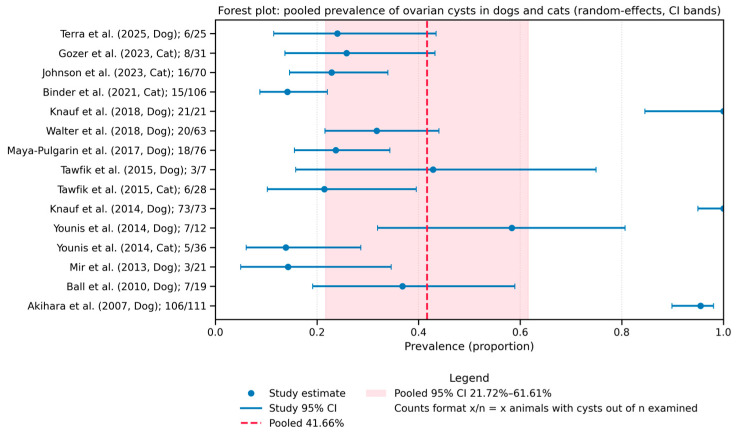
Forest plot of study-specific ovarian cyst prevalence. Each dot shows the study estimate; horizontal lines are Wilson 95% confidence intervals. The dashed vertical line is the pooled random-effects (DerSimonian–Laird) prevalence, and the shaded band indicates its 95% CI. Labels give Author (Year, Species) and cases/total.

The pooled prevalence of ovarian cysts across the 13 included studies was 41.66% (95% CI: 21.72–61.61%), as shown in the forest plot (Figure 3). Heterogeneity between studies was high (I^2^ = 99.1%, τ^2^ = 0.149), indicating substantial variation across the data sources.

Each horizontal line represents a study with corresponding 95% confidence interval. The diamond at the bottom illustrates the overall pooled estimate using a random-effects model (DerSimonian–Laird).

#### 3.4.2. Species Comparison: Dog vs. Cat

The analysis showed a statistically significant difference in the prevalence of ovarian cysts between dogs and cats. Dogs were more likely to develop ovarian cysts compared to cats. The risk ratio (RR) for dogs versus cats was 3.34, and the odds ratio (OR) was 7.12, both with *p* < 0.001 (Fisher exact test and Yates-corrected chi-square test). This suggests that the likelihood of ovarian cysts is significantly higher in dogs than in cats.

#### 3.4.3. Association with Age

**Figure 4:**
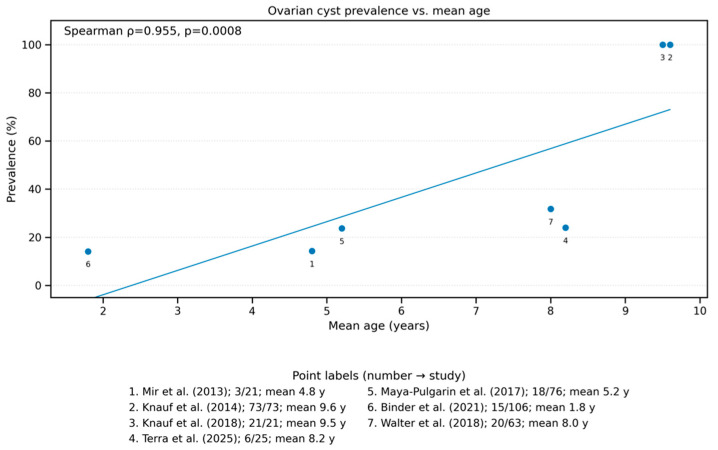
Relationship between the mean age of animals and the prevalence of ovarian cysts. A positive correlation was observed, suggesting that the prevalence of ovarian cysts increases with age. Each dot represents one study reporting both mean age and prevalence data.

There was a strong positive correlation between the mean age of animals and cyst prevalence across studies (Spearman’s ρ = 0.955), indicating that older individuals were more likely to be diagnosed with ovarian cysts. Additionally, linear trend was observed between mean age and prevalence (Figure 4).

#### 3.4.4. Most Common Cyst Types

For the analysis of ovarian cyst types, three studies were excluded—Knauf et al. [4], Gozer et al. [10], and Johnson et al. [11]—due to insufficient information on cyst classification. Based on the remaining studies, the most frequently reported ovarian cysts were follicular cysts, which accounted for 42.1% of all identified cysts (95% CI: 36.4–48.0%, 114/272). Subsurface epithelial cysts were the second most common, representing 37.9% (95% CI: 32.4–43.8%, 103/272), followed by rete ovarii cysts (13.1%, 95% CI: 9.6–17.6%, 35/272) and corpus luteum cysts (6.9%, 95% CI: 4.4–10.5%, 18/272).

### 3.5. Diagnosis

**Figure 5:**
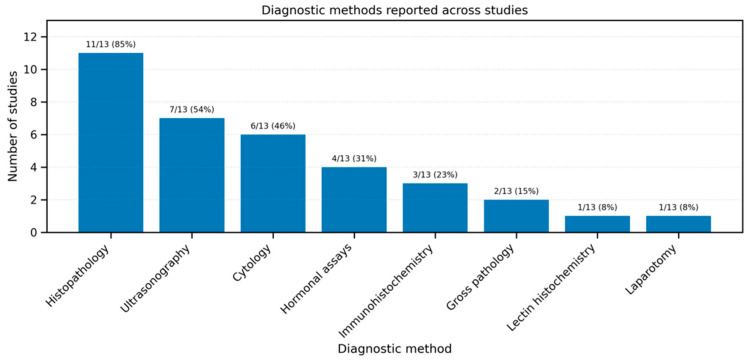
Diagnostic methods used for the identification of ovarian cysts across included studies (*n* = 13).

A variety of diagnostic methods were employed across the included studies to detect ovarian cysts (Figure 5). Histopathology was the most commonly used technique, applied in 11 out of 13 studies, followed by ultrasonography (8 studies) and cytology (7 studies). Immunohistochemistry and hormonal assays (such as AMH, estradiol, or progesterone) were used in 4 studies each, while gross pathology appeared in 2 studies. Laparotomy and lectin histochemistry were reported in individual studies only. These findings highlight the predominance of histopathological and imaging methods in confirming the presence and type of ovarian cysts.

Of the 13 articles, 12 did not describe the specificity or specificity of the diagnostic methods. Nor was there data in the cited literature from which these parameters could be calculated. Only Ball et al. reported that the sensitivity for ultrasound in the diagnosis of cysts was 75% [18].

### 3.6. Treatment of Ovarian Cysts

Systematic research on treatment options, their success rates, and associated side effects for various types of ovarian cysts remains limited. Ovariohysterectomy is generally considered a curative approach in most cases. Moreover, this procedure eliminates the risk of recurrence, particularly in non-breeding females or those affected by severe concurrent uterine pathologies.

Of the literature collected, in all the articles the treatment method was removal of the ovaries, ovarian remnants or the uterus and ovaries. None of the articles reviewed in the meta-analysis undertook drug treatment. Moreover, none of the articles reviewed had data on complications following the procedures.

## 4. Discussion

To the best of our knowledge, this is first meta-analysis performed on such large scale of data. Previously only one meta-analysis about canine cysts has been performed, but only on two studies [20]. This is probably due to the fact that there are few cross-sectional studies published in this area, and a lot of descriptions of individual clinical cases [21,22]. This situation indicates the need for more research on ovarian cysts in bitches and cats. Given the often incidental discovery of cysts during elective castration or necropsy, implementing routine screening protocols, especially in older or breeding animals, could improve early detection and provide more reliable prevalence estimates. In addition, the predominance of retrospective and cross-sectional study designs limits the ability to infer causality. Future prospective cohort studies would allow for a better understanding of the natural history and progression of ovarian cysts in dogs and cats. In clinical practice, ovaries are often not analyzed in detail during OVH. The introduction of systematic macroscopic and histopathological examination of removed ovaries may increase recognition and generate new data in this field.

Another aspect is that more studies on ovarian cysts are conducted on dogs than on cats. This is probably due to the peculiarities of the female cat’s estrous cycle, which influences the faster decision to castrate the female [23]. This situation may make it extremely difficult to collect a large group of mature cats for testing. The ovarian cysts occurs more frequently in older animals than in young ones [2]. In our meta-analysis, while age was significantly associated with higher prevalence of cysts, other potential risk factors such as breed, reproductive status, hormonal treatments, or environmental conditions were not systematically explored across studies. It is important to note that only 7 studies contained data where age-based analysis could be computed, therefore the computed correlation would be subject to change if more data on the subject was available (Figure 4). These areas represent critical gaps that warrant further investigation especially in bitches and cats intended for breeding, cysts can lead to infertility, which is why early diagnosis is particularly important for breeding line planning [1,2].

A notable limitation of the available literature is the lack of data on the sensitivity and specificity of diagnostic methods used to identify ovarian cysts. Although a range of diagnostic techniques were used, including ultrasonography, histopathology, cytology, and hormonal assays, there was no consistent protocol across studies. This heterogeneity highlights the urgent need for standardized diagnostic criteria and protocols for identifying ovarian cysts in companion animals as it takes place in human medicine [24]. There is a lack of clearly defined steps to follow in cases of suspected cysts—it is advisable to develop clinical diagnostic and therapeutic algorithms. In 13 out of 14 studies included in this review, these diagnostic performance parameters were either not reported or not calculable from the available data. Only one study (Ball et al.) provided information on sensitivity, reporting a value of 75% for ultrasonography [18]. This significant gap in the literature highlights the urgent need for well-designed studies evaluating and validating diagnostic tools for ovarian cysts in small animals. An overlooked aspect in many studies on ovarian cysts is the lack of hormonal testing of peripheral blood and cyst fluid. This could be of great value. In animals that are not scheduled for immediate castration, periodic hormone testing (estradiol, progesterone) could be a tool for early detection of hormonally active cysts and further decision-making regarding treatment.

In addition, there is a lack of current research on non-surgical treatment methods probably due to fact that the pathophysiology of ovarian cysts remains unclear. This is particularly important because the problem affects breeding animals, in which surgery to remove the ovaries is associated with loss of fertility and thus the possibility of further reproduction [2].

The overall methodological quality of the included studies was moderate, with the majority rated as having a moderate risk of bias. Only one study met criteria for a low risk of bias, while two were considered at high risk. These findings indicate that caution is needed when interpreting the results of this meta-analysis, as methodological limitations may affect the reliability of reported outcomes.

Future research should focus on prospective, standardized studies assessing both diagnostic accuracy and treatment efficacy. Comparative studies of diagnostic modalities, particularly ultrasonography versus histopathology, could provide valuable insights for clinical decision-making.

## 5. Conclusions

These findings highlight the urgent need for prospective, standardized research focusing on diagnostic accuracy, non-surgical treatment options, and the long-term clinical implications of ovarian cysts in small animals. The most important seems to be that the lack of knowledge of physiopathology as a major limitation for effective medical treatments. Future studies should also consider stratifying data by breed, reproductive status, and hormonal exposure to better understand risk factors and inform evidence-based clinical decisions. This meta-analysis confirms that ovarian cysts are a relatively common finding in bitches and queens, particularly in older animals, but more research in this field is needed.

## Figures and Tables

**Table 1:** Keywords used during literature search.

Base	Keywords
PubMed	(“Ovarian Cysts”[MeSH] OR “ovarian cyst” OR “ovarian cysts”) AND (“dog” OR “bitch” OR “female dog”) AND (“prevalence” OR “diagnosis” OR “ultrasound” OR “treatment” OR “surgery”)
	(“Ovarian Cysts”[MeSH] OR “ovarian cyst” OR “ovarian cysts”) AND (“cat” OR “queen” OR “female cat”) AND (“prevalence” OR “diagnosis” OR “ultrasound” OR “treatment” OR “surgery”)
	Ovarian Cysts[MeSH] OR “ovarian cyst” OR “ovarian cysts”) AND (“dog” OR “bitch” OR “female dog”)
	(“Ovarian Cysts”[MeSH] OR “ovarian cyst” OR “ovarian cysts”) AND (“cat” OR “queen” OR “female cat”)
	canine ovarian cysts treatment
	feline ovarian cysts treatment
Scopus	TITLE-ABS-KEY(“ovarian cyst” OR “ovarian cysts” OR “cystic ovary”) AND TITLE-ABS-KEY(dog OR dogs OR “female dog” OR bitch OR canine OR canines OR cat OR cats OR queen OR queens OR feline OR felines) AND TITLE-ABS-KEY(prevalence OR diagnosis OR ultrasound OR histopathology OR treatment OR therapy OR surgery)
Google scholar	“ovarian cysts” AND (surgery OR “ovariohysterectomy” OR “hormonal treatment”) AND (dog OR bitch)
	“ovarian cysts” AND (surgery OR “ovariohysterectomy” OR “hormonal treatment”) AND (cat OR queen)

**Table 2:** Data from Included Studies.

No.	Author	Year	Country	Species	Number of Animals	Number of Animals with Ovarian Cysts	Follicular Cyst	Corpus Luteum Cyst	Rete Ovarii Cyst	Cysts of Subsurface Epithelial Structures	Other
1	Terra et al. [9]	2025	Brazil	Dog	25	6	3	3	0	0	0
2	Gozer et al. [10]	2023	Türkiye	Cat	31	8	No data	No data	No data	No data	No data
3	Johnson et al. [11]	2023	USA	Cat	70	16	No data	No data	No data	No data	No data
4	Binder et al. [12]	2021	Austria	Cat	106	15	4	1	2	0	8
5	Knauf et al. [1]	2018	Germany	Dog	21	21	32	7	20	37	7
6	Walter et al. [13]	2018	Germany	Dog	63	20	8	7	0	5	0
7	Maya-Pulgarin et al. [14]	2017	Columbia	Dog	76	24	14	0	0	4	0
8	Tawfik et al. [15]	2015	Egypt	Dog	7	3	43	1	1	1	0
				Cat	28	6	21	6		0	0
9	Knauf et al. [4]	2014	Germany	Dog	73	73	No data	No data	No data	No data	No data
10	Younis et al. [16]	2014	Egypt	Dog	12	7	58	7	0	0	0
				Cat	36	5	14	4	0	1	0
11	Mir et al. [17]	2013	France	Dog	21	3	3	0	0	0	0
12	Ball et al. [18]	2010	USA	Dog	19	7	7	0	0	0	0
				Cat	2						
13	Akihara et al. [19]	2007	Japan	Dog	111	106	26	0	12	57	14

## Data Availability

Article The original contributions presented in this study are included in the article/Supplementary Material. Further inquiries can be directed to the corresponding author.

## Supplementary Materials

The following supporting information can be downloaded at https://www.mdpi.com/article/10.3390/ani15192800/s1. Table S1: List of canine and feline breeds involved in the meta analysis.

## Abbreviations

The following abbreviations are used in this manuscript:

AMH	Anti-Müllerian Hormone
CI	Confidence Interval
GnRH	Gonadotropin-Releasing Hormone
hCG	Human Chorionic Gonadotropin
NOS	Newcastle–Ottawa Scale
OR	Odds Ratio
OVH	Ovariohysterectomy
PRISMA	Preferred Reporting Items for Systematic Reviews and Meta-Analyses
PROSPERO	International Prospective Register of Systematic Reviews
RCT	Randomized Controlled Trial
RoB-2	Cochrane Risk of Bias 2
RR	Risk Ratio
